# Functional Magnetic Nanoparticles and Mill Scale for Phosphorus Extraction from Contaminated Water

**DOI:** 10.3390/nano16181150

**Published:** 2026-09-14

**Authors:** Rajpreet Kaur, Mandeep Singh Bakshi

**Affiliations:** Department of Chemistry, Natural and Applied Sciences, University of Wisconsin-Green Bay, 2420 Nicolet Drive, Green Bay, WI 54311-7001, USA; bakshim@uwgb.edu

**Keywords:** phosphorus, magnetic nanoparticles, mill scale, agriculture runoff, surface adsorption

## Abstract

Phosphorus (P) contamination in agricultural runoff is a major environmental concern due to its contribution to eutrophication and deterioration of water quality. Ortho-phosphate extraction from aqueous model systems and agricultural runoff samples was investigated using cetyltrimethylammonium bromide-magnetic nanoparticles (CTAB-MNPs), sodium dodecylsulfate-magnetic nanoparticles (SDS-MNPs), and Mill scale. It was monitored using UV–visible spectroscopy based on the molybdenum blue method, while the adsorption mechanism of P on MNPs was evaluated through time-dependent studies, FTIR, zeta potential (ζ), X-ray photoelectron spectroscopy (XPS), and FESEM-EDS. CTAB-MNPs promoted P adsorption through favorable electrostatic interactions between positively charged quaternary ammonium groups and negatively charged phosphate ions, while SDS-MNPs enhanced P uptake by improving nanoparticle dispersion, colloidal stability, and accessibility of iron oxide active sites. Time-dependent studies revealed that the adsorption kinetics followed the order SDS-MNPs > Mill Scale > CTAB-MNPs. IR, XPS, FESEM-EDS and ζ analyses confirmed the presence of adsorbed P on the surface of MNPs. The results demonstrated that P removal was also governed by inner-sphere complexation with iron oxide active sites rather than electrostatic interactions alone, highlighting the potential of surfactant-modified MNPs and Mill scale as effective materials for P extraction from agricultural runoff.

## 1. Introduction

P is an essential nutrient for agricultural productivity, as excessive P discharge into aquatic systems can lead to serious environmental problems such as eutrophication, algal blooms, and deterioration of water quality [1]. Agricultural runoff, fertilizer leaching, and wastewater effluents are considered major sources of phosphate contamination in natural water resources [2]. Elevated phosphate concentrations accelerate the growth of algae and aquatic plants, which can ultimately result in oxygen depletion and disruption of aquatic ecosystems. Thus, efficient and sustainable removal of phosphate from agricultural runoff has become an important environmental challenge [3,4].

Various techniques are used for P removal, which include chemical precipitation, biological treatment, ion exchange, membrane filtration, and adsorption processes [5,6,7]. Among these approaches, adsorption-based methods have attracted significant attention due to their operational simplicity, cost effectiveness, and high removal efficiency. In particular, iron-based materials have been widely studied because of their strong affinity toward phosphate through surface complexation and precipitation reactions [8,9]. Industrial by-products such as Mill scale (mainly consists of iron oxides, FeO, Fe_3_O_4_, and Fe_2_O_3_) is one of the promising low-cost materials for phosphate removal due to the presence of reactive iron oxide surfaces [10,11].

In recent years, nanomaterials have emerged as highly efficient adsorbents for water purification applications [12]. MNPs, particularly Fe_3_O_4_-based materials, have attracted considerable interest by introducing specific surface charges or functional groups that interact with the material because of the large surface area for adsorption and their inherent ability to respond to an external magnetic field. Surface modification of MNPs with surfactants further enhances their ability to adsorb target pollutants [13,14].

Surfactant-coated MNPs represent a versatile platform for controlling surface interactions with ionic species. For instance, CTAB generates a positively charged surface that promotes electrostatic interaction with phosphate ions. Conversely, SDS produces negatively charged surfaces that may influence phosphate adsorption through electrostatic interactions with surface adsorbed Na^+^ counterions [15]. Understanding surface chemistry that influences phosphate adsorption is important for designing effective nanoparticle-based adsorbents, while engineered nanomaterials and naturally occurring or industrial iron-rich materials such as mill scale offer a sustainable alternative for phosphate removal. Iron oxide phases present in Mill scale provide plenty of active surface sites suitable for the adsorption of phosphate and induce iron–phosphate precipitation [11]. Interestingly, the mechanism and efficiency of phosphate removal by Mill scale differ significantly from those observed for functionalized MNPs.

Here, P extraction is investigated using CTAB-MNPs, SDS-MNPs, and Mill scale. In the aqueous phase, P is predominantly present as dissolved phosphate ions, mainly in the form of orthophosphate such as H_2_PO_4_^−^ and HPO_4_^2−^. We elucidate different mechanisms governing removal through electrostatic adsorption, diffusion-controlled interactions, and iron–phosphate complex formation. The results highlight the significance of surface chemistry in controlling phosphate extraction and demonstrate potential strategies for developing effective materials for P removal from contaminated water and agricultural runoff.

## 2. Materials and Methods

Ferric chloride hexahydrate (FeCl_3_·6H_2_O), ferrous sulfate heptahydrate (FeSO_4_·7H_2_O), ammonium hydroxide solution (NH_4_OH, 35%), cetyltrimethylammonium bromide (CTAB), sodium dodecylsulfate (SDS), and potassium dihydrogen phosphate (KH_2_PO_4_), all more than 99% pure, were obtained from Sigma Aldrich, St. Louis, MO, USA. Mill scale, an iron-rich industrial waste material from Charter steel, Saukville, WI, USA, was collected and used without further chemical treatment. Agricultural runoff samples were collected from surface runoff of a 10-hectare crop field near Kaukauna, WI, USA, at the edge of a field in Wisconsin and supplemented with KH_2_PO_4_ to maintain the P concentration.

### 2.1. Preparation of Blue-Color Phosphate Solution (Molybdenum Blue Method)

P concentration in aqueous solutions was determined by using the molybdenum blue colorimetric method [16]. A known volume of phosphate solution was taken in a clean glass container. A color-developing reagent was added sequentially under continuous mixing. In an acidic medium, phosphate reacts with molybdate ions to form phosphomolybdic acid that produced blue-colored complex. The solution was allowed to stand for sufficient time to ensure complete color development. The intensity of the blue color was directly proportional to the phosphate concentration present in the solution and was visually observed for comparative analysis of phosphate removal before and after adsorption experiments.

### 2.2. Synthesis of CTAB- and SDS-MNPs

Water-soluble iron oxide nanoparticles were prepared using a hydrothermal synthesis approach reported previously [17]. In a typical procedure, a 20 mL reaction solution containing FeCl_3_ (4 mM) and FeSO_4_ (4 mM) in a 1:1 molar ratio was prepared, followed by the addition of 250 mg of CTAB. The reaction was initiated by adding 35% ammonium hydroxide, which acted as a base catalyst. The resulting mixture was transferred to a Teflon-lined container, sealed within a steel autoclave, and maintained at 150 °C for 24 h. After completion of the reaction, the MNPs were separated using an external magnet and repeatedly washed with distilled water to remove excess CTAB. SDS produced surface active SDS-MNPs with similar procedure. They were insoluble in water, purification was carried out using hexane, and the final product was stored in hexane for further use. The microscopic images of both samples are shown in the Supplementary Materials (Figure S1).

### 2.3. Characterization of Real Agricultural Runoff

The agricultural runoff used in this study was characterised based on its pH, electrical conductivity, and phosphorus concentration to establish its physicochemical and nutrient characteristics. These parameters provided a representative baseline for evaluating the phosphate-removal performance of the synthesized functional MNPs and Mill scale under realistic runoff conditions (Table S1).

### 2.4. UV–Vis Titration Experiment

UV–Vis spectrophotometric titration experiments were performed to evaluate the progressive removal of phosphate by the functionalized magnetic nanoparticles. For the standard phosphate solutions, KH_2_PO_4_ was used as the phosphate source, and phosphate concentrations of 0.25, 0.50, and 1.00 mg P L^−1^ were investigated. In each experiment, 3.0 mL of the phosphate solution was directly transferred into a UV–Vis cuvette. For CTAB-MNPs, a 20 mM CTAB-MNP suspension was added stepwise to the phosphate solution, and the cumulative CTAB-MNP concentrations were increased progressively with each addition. Similarly, an 80 mM SDS-MNP suspension was added stepwise to a 1.00 mg P L^−1^ phosphate solution; the corresponding cumulative SDS-MNP concentrations were increased progressively during titration. After each addition of MNPs, a magnet was immediately placed adjacent to the cuvette to collect the magnetic nanoparticles at the cuvette wall. No shaking, stirring, vortexing, or other external agitation was applied during the experiment. Following magnetic separation, the UV–Vis spectrum of the clarified solution was recorded. The interval between MNP addition, magnetic separation, and spectral acquisition was approximately 1–2 min and represents the practical handling and magnetic separation time rather than a separately controlled adsorption contact time. The stepwise addition of MNPs and spectral measurements were continued until the characteristic blue color and corresponding absorption bands of the molybdenum-blue phosphate complex were substantially diminished. In addition to the KH_2_PO_4_ standards, a 0.50–0.65 mg P L^−1^ agricultural runoff sample was also investigated under the same spectrophotometric titration procedure, where the phosphate concentration in the form of O-phosphate of the untreated runoff (unfiltered and undigested) was independently determined using an AQ-300 analytical instrument (SEAL Analytical, Inc., Mequon, WI, USA) [18]. These experiments enabled the spectral response of O-phosphate-containing solutions to be monitored progressively with increasing concentrations of CTAB-MNPs and SDS-MNPs.

### 2.5. Time-Dependent Experiment

Time-dependent phosphate removal experiments were performed using CTAB-MNPs, SDS-MNPs, and Mill scale. For each experiment, 3.0 mL of a 0.5 mg P L^−1^ phosphate solution or agricultural runoff containing an experimentally determined phosphorus concentration of 0.5 mg P L^−1^ was transferred into a UV–Vis cuvette. Based on the preceding titration experiments, the adsorbent amounts used for the time-dependent studies were selected as 1.0 mL of 20 mM CTAB-MNP suspension and 1.0 mL of 80 mM SDS-MNP suspension, whereas 1.0 g of Mill scale was added to the phosphate-containing solution with a 1:3 solid–liquid ratio. Immediately after addition of the respective adsorbent, the samples were kept undisturbed under static conditions at room temperature, without shaking, stirring, or any other external agitation. UV–Vis spectra were subsequently recorded at predetermined time intervals over a total period of 0–1200 min to monitor the time-dependent change in phosphate-related absorbance. The same experimental procedure was followed for CTAB-MNPs, SDS-MNPs, and Mill scale, allowing for comparison of their phosphate-removal behavior as a function of contact time.

### 2.6. Characterization and Sample Analysis

UV−visible measurements of blue-colored phosphate solutions before and after the extraction were carried out using a UV−visible instrument (Shimadzu-Model No. 2450, double beam; Shimadzu Corporation, Kyoto, Japan) equipped with a TCC 240A thermoelectrically temperature-controlled cell holder. Fourier transform infrared spectroscopy (Nicolet ID5 ATR; Thermo Fisher Scientific, Madison, WI, USA) with a wavenumber range of 4000 cm^−1^ to 500 cm^−1^ was used to determine P sorption before and after the treatment with surfactant-coated magnetic nanoparticles and Mill scale. X-ray photoelectron spectroscopy (XPS) analysis was performed using a PHI ULVAC VersaProbe 4 system (ULVAC-PHI, Kanagawa, Japan) equipped with a scanning monochromatic Al Kα X-ray source (1486.6 eV, 100 µm beam diameter) operated at 25 W and 15 kV, with pass energies of 224 eV for survey scans and 55 eV for high-resolution scans for surface analysis of samples. X-ray diffraction (XRD) patterns were characterized by using a Bruker-AXS D8-GADDS instrument (Bruker AXS GmbH, Karlsruhe, Germany) with Tsec = 480. Samples were prepared on glass slides by spotting a concentrated drop of aqueous suspension and dried in a vacuum desiccator. Field-emission scanning electron microscopy analyses (FESEM) were carried out by dispersing a drop on a carbon-tapped stub, coated with platinum, and then analyzed with a Hitachi SU8010-SE instrument (Hitachi High-Tech Corporation, Tokyo, Japan) with a resolution of 1.3 nm at a landing voltage of 1.0 kV. EDS analyses were also performed simultaneously by a Bruker eds model XFlash 6130 instrument (Bruker Nano GmbH, Berlin, Germany) with a peak resolution of 121 eV.

## 3. Results and Discussion

### 3.1. P Removal with CTAB-MNPs

**Figure 1:**
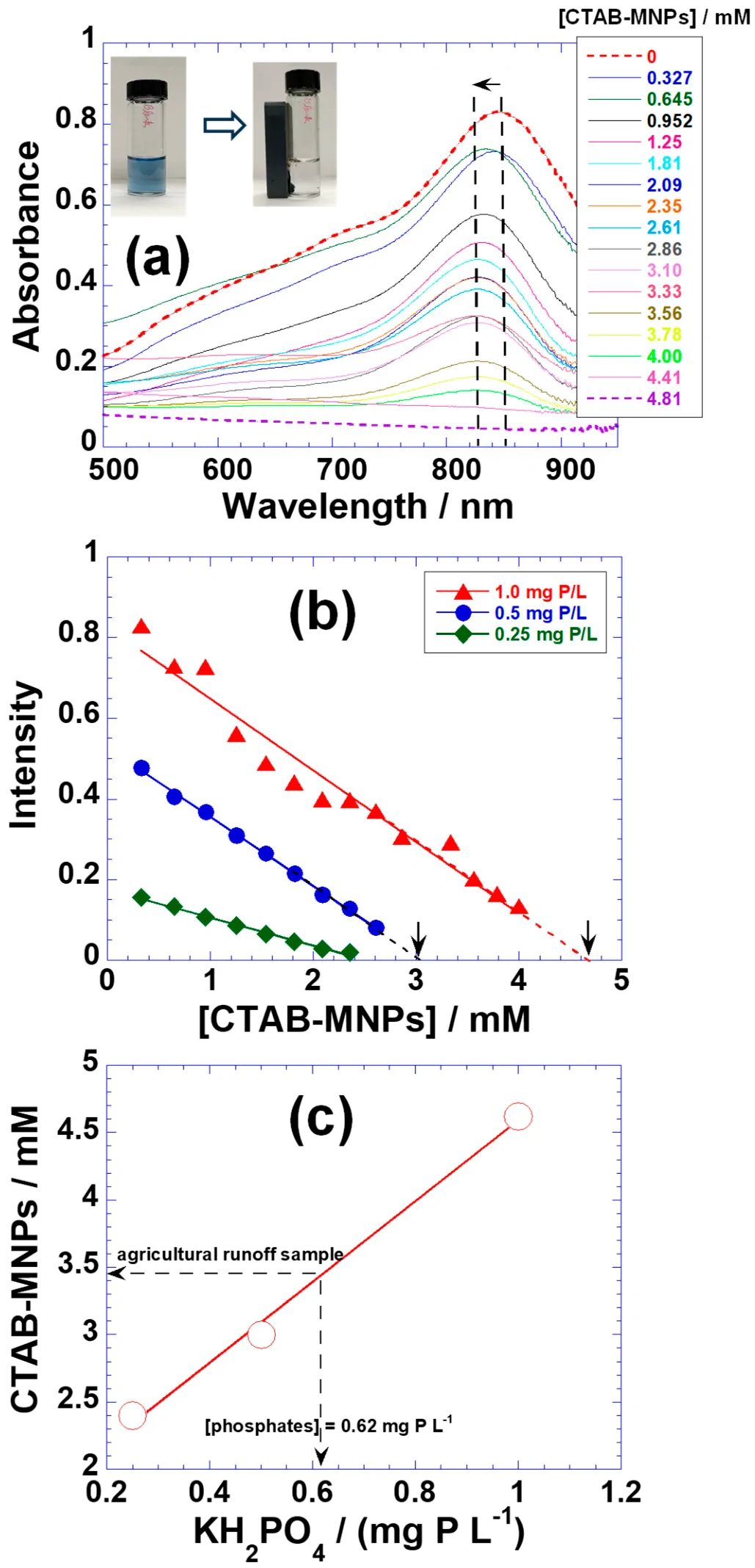
(**a**) UV-visible titration profiles showing P removal from aqueous KH_2_PO_4_ solution (1 mg P L^−1^) using CTAB-MNPs. A progressive decrease in absorbance at ~880 nm indicates adsorption of P onto CTAB-MNPs. (**b**) Variation in intensity of P in different phosphate aqueous model solutions versus the amounts of CTAB-MNPs. (**c**) Standard plot between amounts of CTAB-MNPs and KH_2_PO_4_.

Initially, the P model aqueous system is chosen to understand the extraction by MNPs. UV–visible spectrum of the molybdenum blue complex exhibits a broad absorption band centered around ~850 nm (Figure 1a), which is the characteristic feature of the reduced phosphomolybdate complex used for P determination [16]. During stepwise addition of CTAB-MNPs, a gradual decrease in absorbance occurs, indicating a progressive decrease in the concentration of phosphorus-containing species remaining in the aqueous phase after magnetic separation [13,14,18]. A blue shift of ~20 nm in the absorption of P in form of O-phosphate suggests a change in the electronic environment of the molybdenum blue complex as the concentration of free phosphate ions decreases in the bulk. The interactions between P and positively charged CTAB-MNPs alter the coordination environment of the phosphomolybdate complex with a depleting amount of phosphates in the aqueous bulk. The intensity–concentration relationship (Figure 1b) further demonstrates the progressive decrease in aqueous-phase phosphorus with increasing CTAB-MNP concentration. A linear decrease in the absorbance intensity for all O-phosphate concentrations (0.25–1 mg P L^−1^) with comparable slopes indicates efficient P removal through electrostatic interactions between negatively charged phosphate ions and the cationic CTAB-MNPs. It also suggests that MNPs bear plenty of active sites available on the solid–liquid interface adsorption of P and are equally involved in the electrostatic adsorption and extraction of P from the aqueous bulk. The extrapolation of each plot to zero intensity (Figure 1b, indicated by black arrows) depicts the concentration of CTAB-MNPs required for the complete extraction of P from the aqueous bulk. The required amount of CTAB-MNPs for the complete removal of P varies linearly and can be used as a standard plot (Figure 1c) to determine the concentration of P in unknown real agricultural samples.

**Figure 2:**
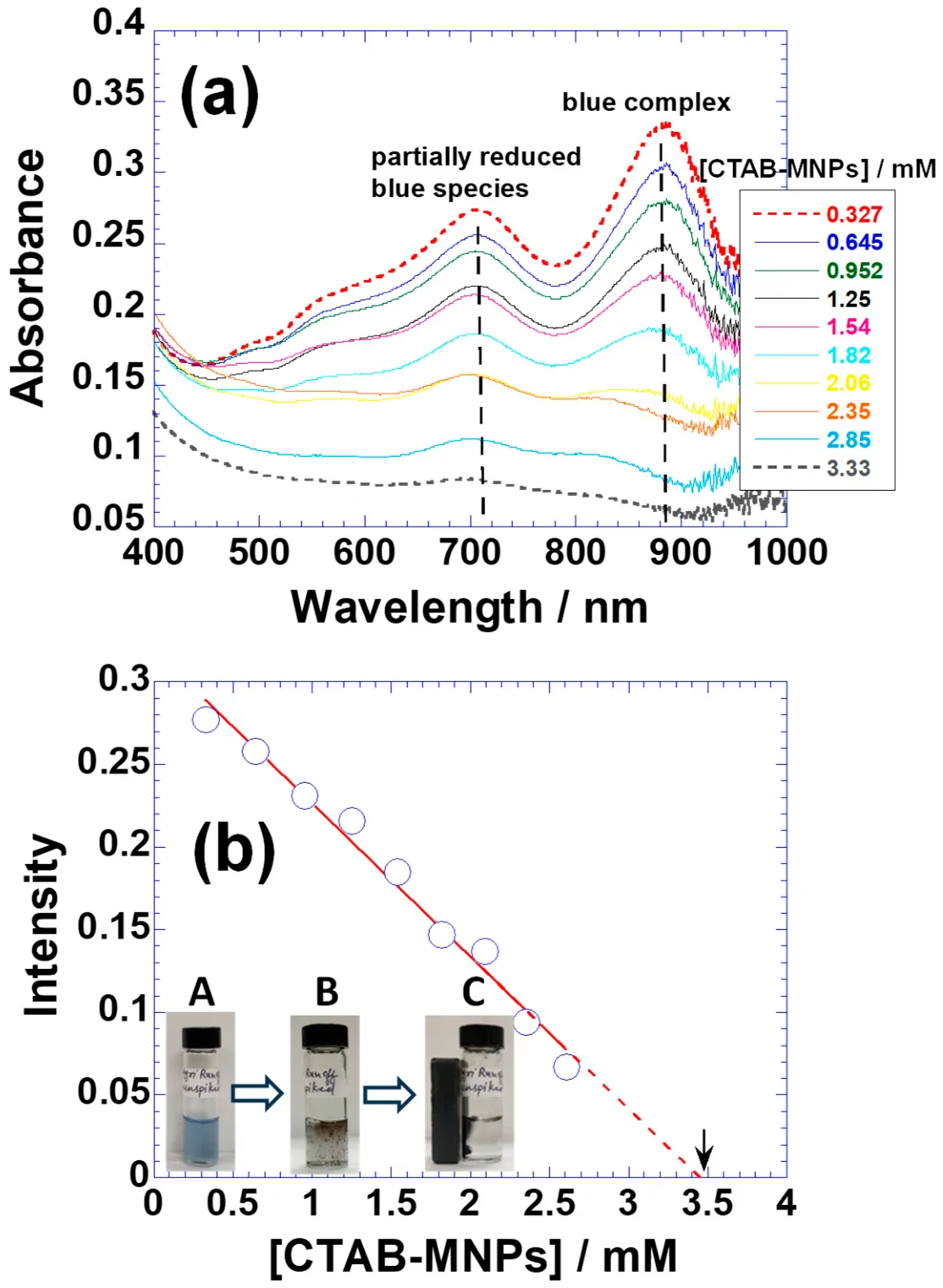
(**a**) UV–visible titration of P removal from real sample of agricultural runoff using CTAB-MNPs. The spectra display two characteristic absorption bands at ~700 nm and ~880 nm corresponding to molybdenum blue species. Progressive decrease in absorbance intensity during titration indicates adsorption and extraction of P from the solution. (**b**) Linear variation in intensity versus concentration of CTAB-MNPs. Photographic images (A–C) illustrating P extraction from agricultural runoff using CTAB-MNPs. Initial colored sample after molybdenum blue development (A), aggregation of CTAB-MNPs during titration (B), and magnetic separation of P-extracted CTAB-MNPs leaving behind transparent clear solution without MNPs (C).

Extraction of P from real agricultural runoff sample exhibits two characteristic absorption bands. A dominant peak around 880 nm corresponds to the molybdenum blue phosphomolybdate complex, while a secondary band around 700 nm is attributed to the partially reduced polymolybdate species formed during the color development reaction [16]. The additional band observed in the real runoff may be associated with the more complex chemical matrix of the agricultural runoff and the presence of co-existing dissolved constituents that can influence the molybdate reduction and speciation during color development (Figure 2a). A stepwise addition of CTAB-MNPs decreases the intensity of both bands (Figure 2b), indicating a progressive decrease in the phosphorus-containing species remaining in the aqueous phase after magnetic separation. The extrapolation of this plot to zero intensity (indicated by black arrow) determines the amount of CTAB-MNPs required for complete P removal from the agricultural sample. The extracted P concentration in the real agricultural runoff sample was thus determined from the standard plot (Figure 1c) as 0.62 mg P L^−1^, which agrees well with the dissolved P concentration of 0.65 mg P L^−1^ independently determined using the AQ-300 Discrete Analyzer (SEAL Analytical GmbH, Norderstedt, Germany) following the U.S. EPA Method 118-D Rev 1.

### 3.2. P Removal with SDS-MNPs

P extraction with SDS-MNPs displays two characteristic absorption bands of the molybdenum blue complex (Figure S2), as noted above with CTAB-MNPs. SDS-MNPs do not show a marked decrease in the absorbance intensity, indicating limited interactions between P species and SDS-MNPs due to the presence of anionic sulfate head groups of SDS [15,17]. In fact, hexane- soluble SDS-MNPs do not incorporate into the aqueous bulk; rather, these stay in the hexane layer above the aqueous phase. However, the extraction of P happens through the aqueous–hexane interface while leaving the sample undisturbed overnight, thereby reducing the absorbance of molybdenum blue complex [19,20]. The last concentration of SDS-MNPs = 6.33 mM in Figure S2 when repeated after two days, the characteristic absorption bands reduce to minimum. This shows that the extraction occurs through slow diffusion-controlled physisorption rather than electrostatic interactions [19,21]. Time–dependent extraction is discussed later in this section.

### 3.3. P Removal with Mill Scale

**Figure 3:**
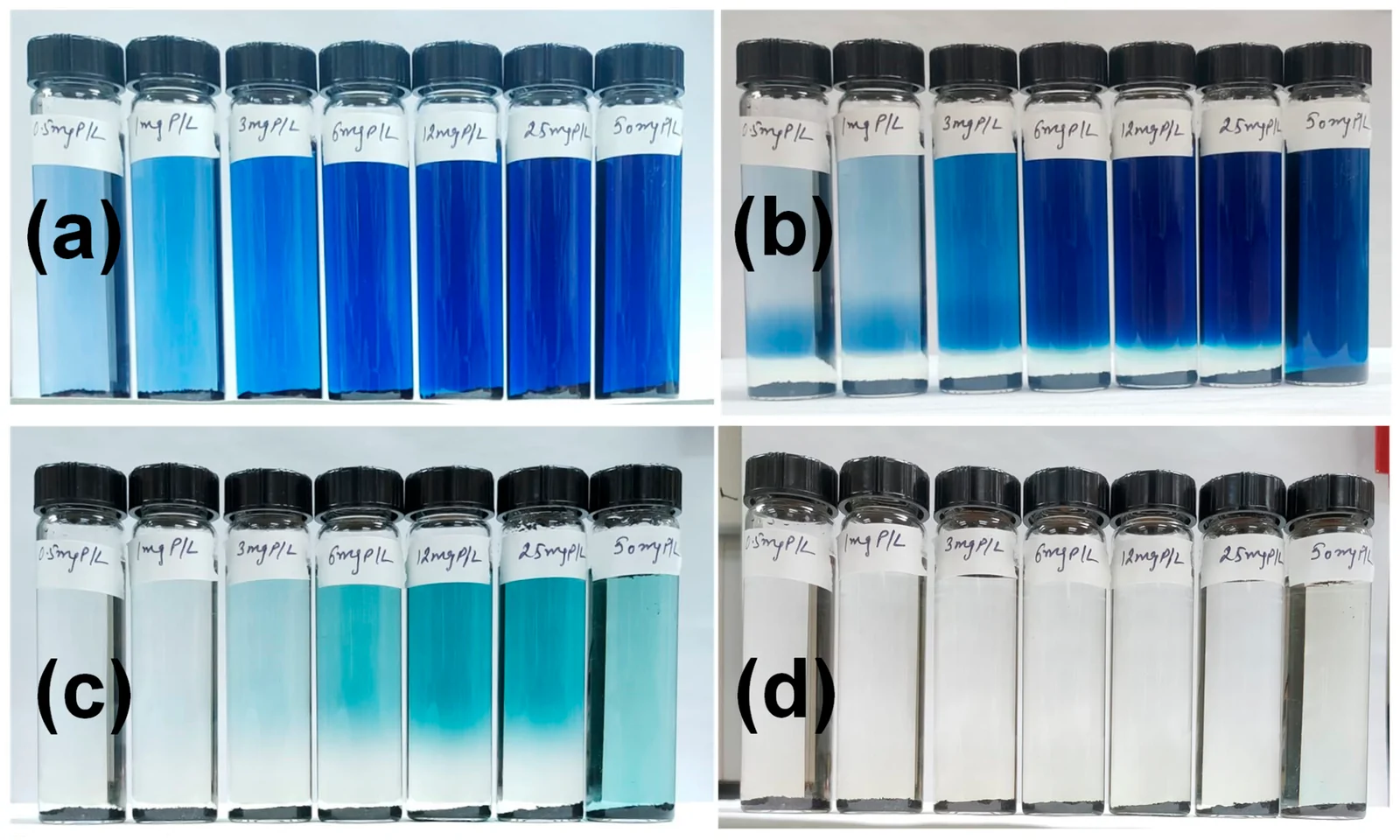
Direct visual observation of P removal using 1 g Mill scale from aqueous phosphate solutions in the concentration range of 1–50 mg P L^−1^. The blue color corresponds to the molybdenum blue phosphate complex. Progressive discoloration of the solution from the 1st to the 6th day (**a**–**d**) indicates gradual P extraction.

As mentioned in the experimental section, Mill scale is an iron-rich industrial waste electrochemically cathodic material primary FeO, Fe_3_O_4_, and Fe_2_O_3_ [22,23]. The XRF analysis of Mill scale used in this study further confirmed its iron-rich oxide composition, with Fe_2_O_3_ as the major constituent (33.25 wt.%), accompanied by substantial amounts of CaO (19.75 wt.%) and SiO_2_ (25.05 wt.%). MgO (6.65 wt.%), Al_2_O_3_ (1.71 wt.%), MnO (0.27 wt.%), and other minor oxide constituents were also detected, whereas P_2_O_5_ was present only at 0.03 wt.%. The substantial Fe- and Ca-containing oxide fractions may provide favorable surface sites and phases for phosphate interaction and immobilization, supporting the potential of Mill scale for phosphorus removal. It bears several active sites for facilitated adsorption of P through redox reactions [10,22]. Unlike the functionalized MNPs mentioned above, their grain size is much larger than MNPs and varies from micron to mm range. Hence, it does not produce colloidal aqueous suspension and cannot be used as titrant like MNPs. Figure 3 shows the phosphate removal ability of Mill scale evaluated through direct visual observation using a phosphate solution in the concentration range of 1–50 mg P L^−1^ facilitated by the molybdenum blue complex over a period of 6 days. The intense blue color of the molybdenum blue complex slowly turns colorless [10,24] after the addition of 1 g of Mill scale due to the adsorption and extraction of P on Mill scale.

### 3.4. Time-Dependent P Removal

**Figure 4:**
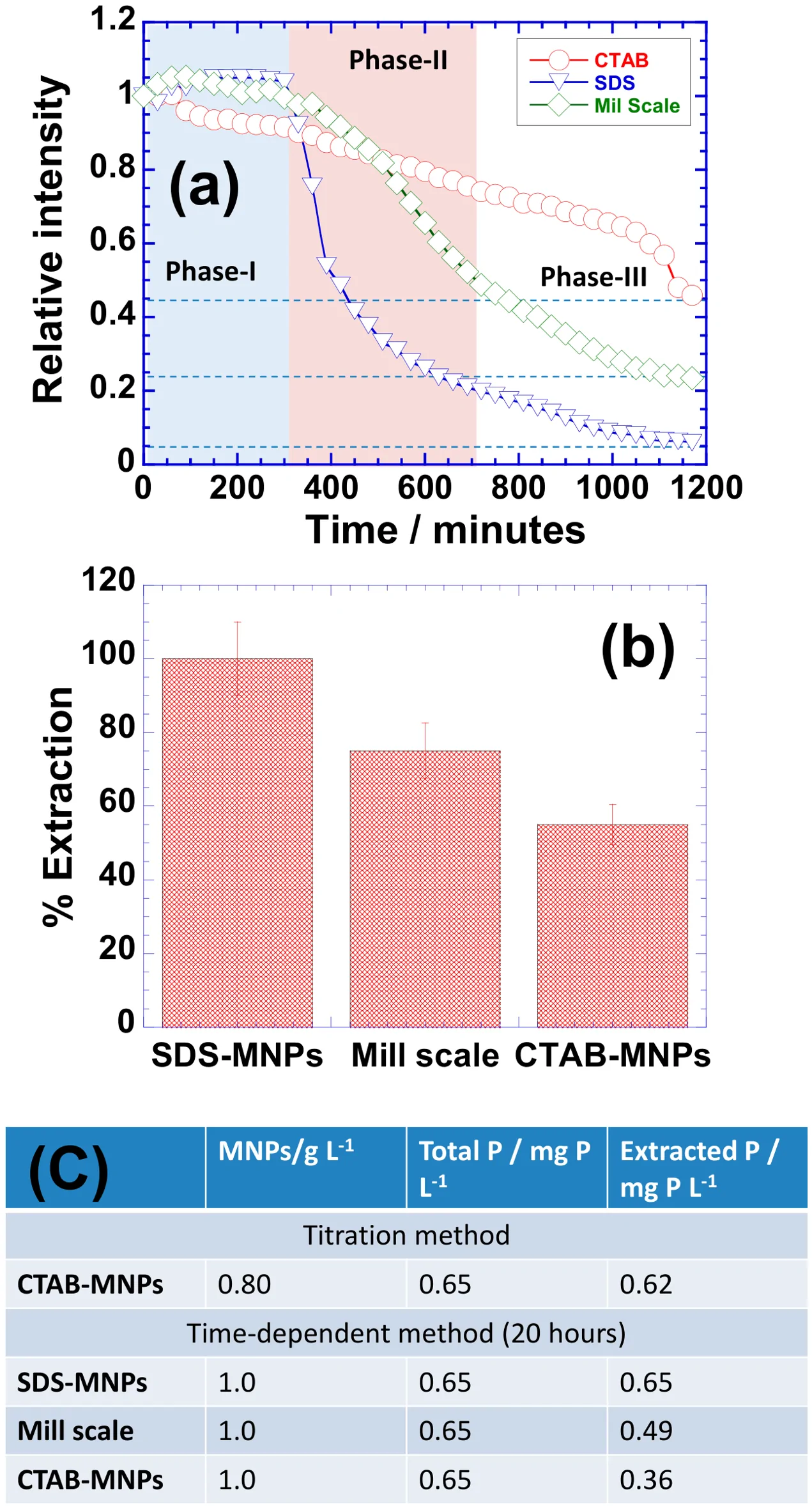
(**a**) Time-dependent profiles of P removal from a real agricultural runoff sample using CTAB-MNPs, SDS-MNPs, and Mill scale. (Phase-I) initial stage of interactions, (Phase-II) diffusion-controlled adsorption, and (Phase-III) equilibrium stage. Dotted lines indicate where intensities of respective profiles reach equilibrium. (**b**) Bar plots of % extraction of CTAB-MNPs, SDS-MNPs, and Mill scale. (**c**) Quantitative extraction of P from aqueous agricultural runoff using titration and time-dependent studies. Error bars here represent the standard deviation of three independent experiments (*n* = 3).

Time-dependent P removal from a real agricultural runoff sample with CTAB-MNPs, SDS-MNPs, and Mill scale is presented in Figure 4a, where a gradual removal of P from aqueous bulk is depicted by a time-dependent decrease in the intensity. Figure 4a is better understood by dividing the variation into three characteristic stages, i.e., initial interactions between the P and active sites available on the solid–liquid interface of MNPs (Phase-I), diffusion-controlled adsorption (Phase-II), and final equilibrium (Phase-III) [21]. The initial interactions at the interface take place within ~300 min, where no significant change is observed among all three cases (Phase-I). Thereafter, a rapid fall in the intensity occurs due to the diffusion of complexed P from aqueous bulk to the interface (Phase-II), which is very much pronounced for SDS-MNPs and Mill scale in comparison to CTAB-MNPs. This demonstrates that different mechanisms of interfacial adsorption of P on SDS-MNPs/Mill scale occur in comparison to that on CTAB-MNPs.

A slight variation in intensity of P for CTAB-MNPs in all three regions suggests that the extraction and adsorption of P on MNPs are primarily governed by the electrostatic interactions between predominantly negatively charged P and positively charged CTAB-MNPs [18]. In other words, aqueous solubilized P is electrostatically attracted and diffused to the interface under the effect of oppositely charged electrostatic interactions and that induces a regular fall in intensity. But extraction is more prominent and rapid when it happens through specific interactions between the surface-active sites available on SDS-MNPs/Mill scale and aqueous- dissolved P [21]. This implies that P extraction is more fruitful when it takes place through diffusion and surface adsorption rather than predominantly under the effect of electrostatic interactions because the surface coating of CTAB on MNPs in fact screens the surface-active sites available for P adsorption [17,18]. This effect seems to be less prominent in the case of SDS-MNPs and that is why it behaves like Mill scale with unmodified surface. Since the time-dependent extraction of P was carried out under static conditions, in contrast to the titration method performed in Figure 2, the amount of extracted P was quantified from the decrease in the characteristic absorbance intensity with time by using Equations (1) and (2).

(1) $$q_{t}=\left[\left(C_{0}-C_{t}\right)V\right]/m$$

where q_t_ is the amount of P extracted at time (mg P g^−1^), and C_0_ and C_t_ are the initial and remaining P concentrations (mg P L^−1^), respectively, V is the volume of the phosphate solution (L), and m is the mass of adsorbent (g).

P removal (%) = [(C_0_ − C_t_)/C_0_] × 100 (2)

where C_0_ and C_t_ represent the initial and remaining P concentrations (mg P L^−1^), respectively.

At 20 h, SDS-MNPs removed almost the entire amount of P, corresponding to 0.65 mg P L^−1^ (100% removal), whereas Mill scale removed approximately 0.49 mg P L^−1^ (75.4% removal) and CTAB-MNPs removed 0.36 mg P L^−1^ (55.4% removal) (Figure 4b). Considering an adsorbent concentration of 1.0 g L^−1^, these values correspond to phosphorus uptakes of 0.65, 0.49, and 0.36 mg P g^−1^ for SDS-MNPs, Mill scale, and CTAB-MNPs, respectively, at 20 h. These values are reported as phosphorus uptake at 20 h (q_20_h) rather than definitive equilibrium adsorption capacities since the present experiment was designed as a time-dependent extraction study rather than a systematic equilibrium adsorption experiment.

A relative comparison between the titration and time-dependent methods in terms of the amount of MNPs used for the P extraction from agricultural runoff sample is depicted in Figure 4c. However, it is not complete because the titration method is only applied with CTAB-MNPs and is not possible to use for SDS-MNPs as well as for Mill scale. This demonstrates that for CTAB-MNPs, the titration method is more efficient in comparison to the time- dependent method, whereas for SDS-MNPs and Mill scale, the time-dependent method is a useful technique.

### 3.5. pH Effect

The pH of agricultural runoff sample is usually strongly alkaline (pH ~10) (Table S1) due to the presence of carbonate and bicarbonate ions derived from soil minerals and residual phosphate-based fertilizers. The dissolution of carbonate minerals (e.g., CaCO_3_) and phosphate fertilisers generate basic species, including CO_3_^2−^ and HPO_4_^2−^, which increases the alkalinity of agricultural runoff [24]. The extraction of P in highly alkaline conditions of agricultural runoff sample becomes highly prominent due to an instant adsorption of P on the surface of CTAB-MNPs. It induces rapid aggregation of MNPs, which allows for an easy separation of P- loaded MNPs using an external magnet (sample photos in Figure 2b). P ions predominantly exist in the forms of HPO_4_^2−^ and PO_4_^3−^, which interact strongly with the positively charged quaternary ammonium groups of CTAB MNPs. In alkaline conditions, the interactions even become much stronger with P ions (Equation (3)). A high ionic strength of the agricultural runoff impedes the electrostatic stabilization of the MNPs, thereby leading to aggregation.

(3) $$Fe_{3}O_{4}-CTAB^{+}+H_{2}PO_{4}^{-}\to Fe_{3}O_{4}–CTAB^{+}\;\to \;Fe_{3}O_{4}–CTAB^{+}+HPO_{4}^{2-}\to Fe_{3}O_{4}–CTAB^{+}\cdots HPO_{4}^{2-}$$

Although, the adsorption of P on SDS-MNPs/Mill scale in alkaline agricultural runoff is quite prominent and rapid (Figure 4), it does not induce a similar degree of self-aggregation among the MNPs due to the lack of charge neutralization, which happens for CTAB-MNPs. It is primarily driven by surface adsorption on oxygen-terminal functional groups of SDS-MNPs/Mill scale (Equation (4)).

(4) $$(Fe_{3}O_{4}–SDS)/Mill\;scale+H_{2}PO_{4}^{-}\to surface\;adsorption\to (Fe_{3}O_{4}–SDS)/Mill\;scale\ldots H_{2}PO_{4}^{-}$$

### 3.6. Zeta Potential (ζ)

ζ provides clear evidence of adsorption of negatively charged phosphate species onto the adsorbent surfaces (Figure S3). P adsorption causes a shift in the ζ potential towards more negative values, decreasing from +19.97 to +9.04 mV for CTAB-MNPs, from −22.02 to −32.82 mV for SDS-MNPs, and from −5.63 to −17.11 mV for Mill scale. The negative shift of ~10 mV confirms the successful adsorption of P on the solid–liquid interface of MNPs. P adsorption is expected to be governed predominantly by ligand exchange as well as Fe–O–P surface complex formation rather than electrostatic interactions alone [10]. The rapid adsorption exhibited by SDS-MNPs/Mill scale (Figure 4a) is attributed to the improved dispersion and accessibility of iron oxide active sites provided by surfactant modification, whereas the negative shift in ζ reflects the increase in surface coverage of P on MNPs [25].

### 3.7. XRD Analysis

**Figure 5:**
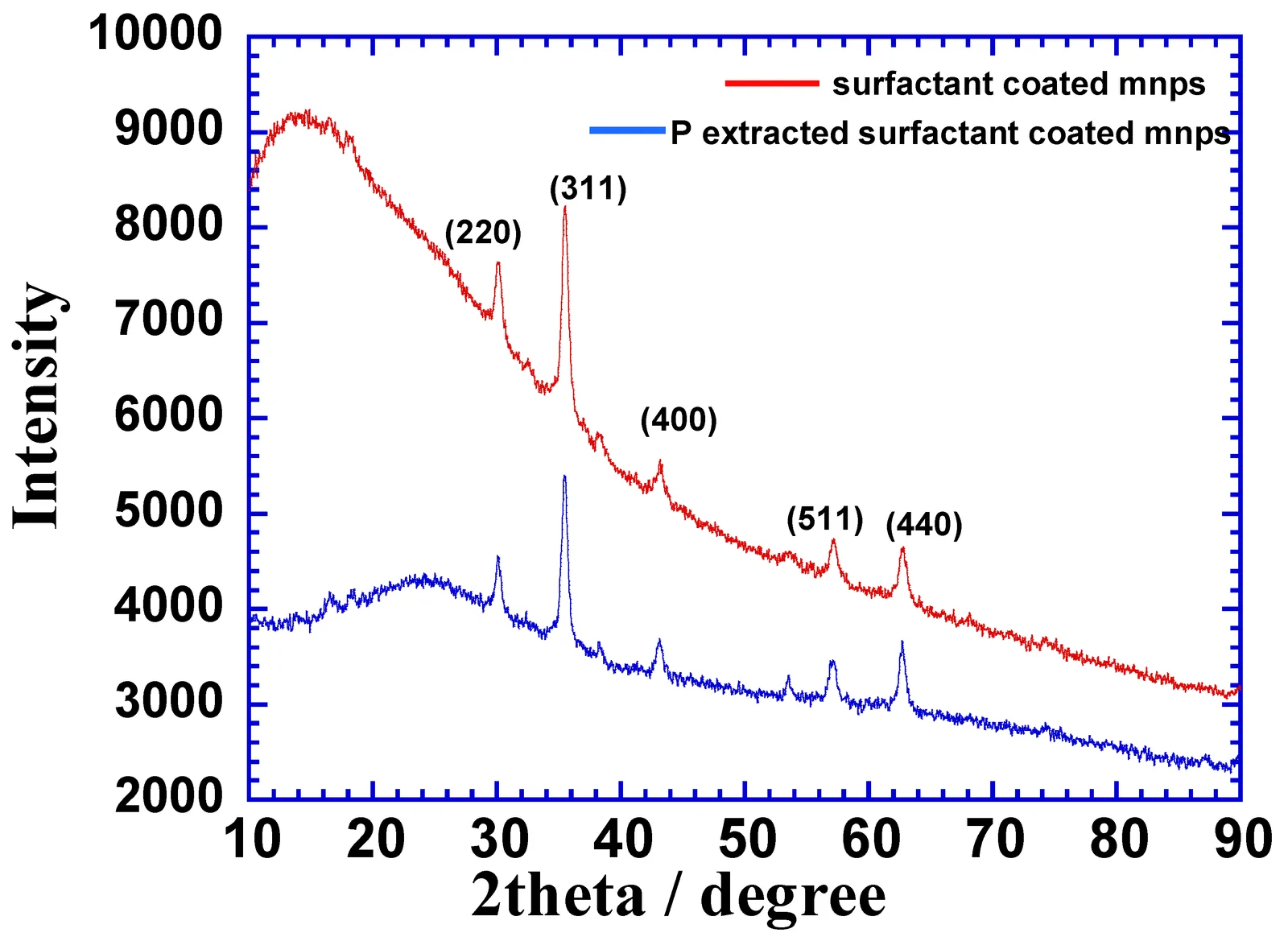
XRD of CTAB-MNPS and P-extracted CTAB-MNPs.

X-ray diffraction (XRD) patterns of the surfactant-coated magnetic nanoparticles (MNPs) before and after phosphorus (P) extraction (Figure 5) confirm the retention of the highly crystalline cubic spinel structure at approximately 30.1°, 35.6°, 43.1°, 53.5°, 57.0°, and 62.6°, which can be indexed to the (220), (311), (400), (422), (511), and (440) crystallographic planes, respectively. The characteristic peak positions (2θ) were retained after P extraction, indicating that the iron oxide core remained structurally stable and did not undergo detectable phase transformation during the extraction process [26]. Furthermore, the absence of additional distinct crystalline reflections in the P-extracted sample (blue curve) suggests that the extracted phosphorus (HPO_4_^2−^) did not form a separately detectable crystalline phosphate phase and was likely associated with the MNP surface in a poorly crystalline or amorphous form. Concurrently, the noticeable modification in the broad amorphous halo in the 10° to 30° region may indicate reorganization of the surfactant-associated surface environment following phosphate interaction [27].

### 3.8. IR Analysis

**Figure 6:**
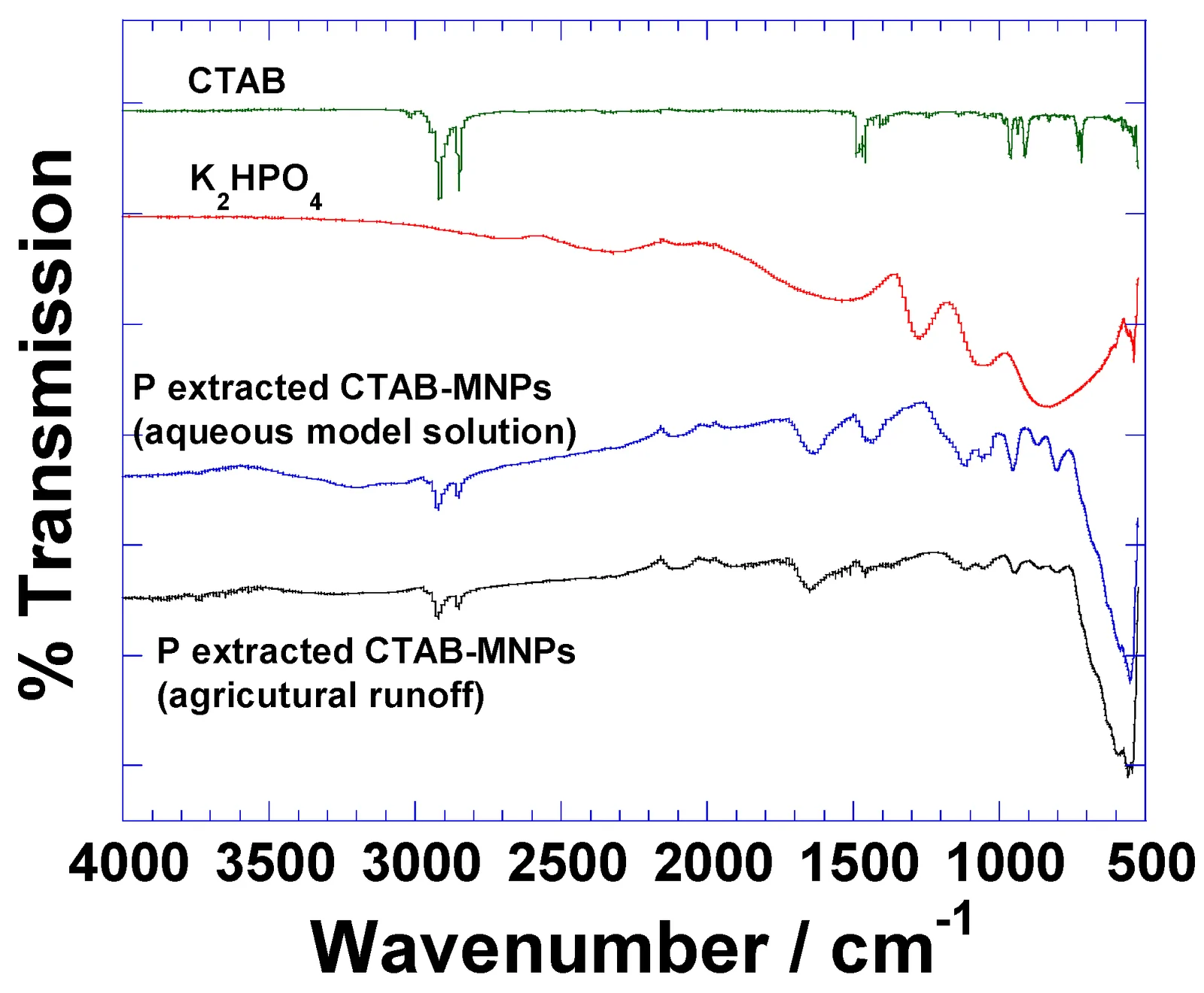
FTIR spectra of pure CTAB, KH_2_PO_4_, P-extracted CTAB-MNPs from an aqueous model solution, and from a real sample of agricultural runoff. The appearance of phosphate stretching vibrations in the region of 1000–1100 cm^−1^ together with Fe–O bands near 500–600 cm^−1^ confirms adsorption of P onto CTAB-MNPs.

IR spectra of pure CTAB and KH_2_PO_4_ are compared with P extracted from the model phosphate solution as well as from agricultural runoff samples in Figure 6 (Table S2a,b). Strong absorption peaks observed near 2943 cm^−1^ and 2870 cm^−1^ correspond to asymmetric and symmetric stretching vibrations of CH_3_ groups, while peaks near 2917 cm^−1^ and 2849 cm^−1^ refer to asymmetric and symmetric stretching vibrations of CH_2_ groups in the hydrocarbon chain. A band near 1468 cm^−1^ corresponds to CH_2_ scissoring vibrations, and additional bands near 1394–1360 cm^−1^ are attributed to alkyl chain deformation modes. These peaks confirm the presence of CTAB molecules associated with the MNPs’ surface [28]. The IR spectrum of pure KH_2_PO_4_ shows characteristic phosphate vibrations in the region of 1000–1100 cm^−1^, which correspond to P–O stretching vibrations of phosphate tetrahedron, while bands near 900–980 cm^−1^ are attributed to P–OH stretching modes. Additional bands observed near 520–540 cm^−1^ correspond to Fe-O-P/O–P–O bending vibrations [29].

The P-extracted CTAB-MNP sample shows phosphate-related bands in the region of 1000–1100 cm^−1^, confirming the presence of adsorbed P. These bands (Table S2a) exhibit a slight shift toward lower wavenumbers and broadening compared with that of the pure phosphate spectrum. The presence of characteristic P–O stretching vibrations, together with Fe–O lattice vibrations, confirms the association of phosphate species with CTAB-MNPs. Similarly, the P-extracted SDS-MNPs’ spectrum (Figure S4) (Table S2b) depicts the coexistence of sulfate and phosphate bands in the 1000–1100 cm^−1^ region [29,30]. The sulfate stretching bands remain largely unchanged for pure SDS and SDS-MNPs, while the phosphate bands appear relatively weaker compared with those observed for P-extracted CTAB-MNPs, suggesting much stronger surface adsorption on SDS-MNPs. A strong adsorption of surfactant promotes hydrophobic interactions among the surfactant molecules, thereby diminishing their IR modes [29]. IR spectrum of P-extracted Mill scale (Figure S5) mainly highlights the characteristic iron oxide vibrations near ~560 cm^−1^, corresponding to Fe–O stretching modes of iron oxide phases present in Mill scale. All other bands related to P adsorption are very weak due to the large grain size of Mill scale. The FTIR findings complement the XRD data by explicitly verifying that while the structural integrity of the magnetic core remains unaltered, the phosphorus extraction proceeds successfully via high-affinity chemical binding of phosphate species onto the surfactant-modified surface matrices.

### 3.9. Surface Analysis

*FESEM-EDS analysis*

**Figure 7:**
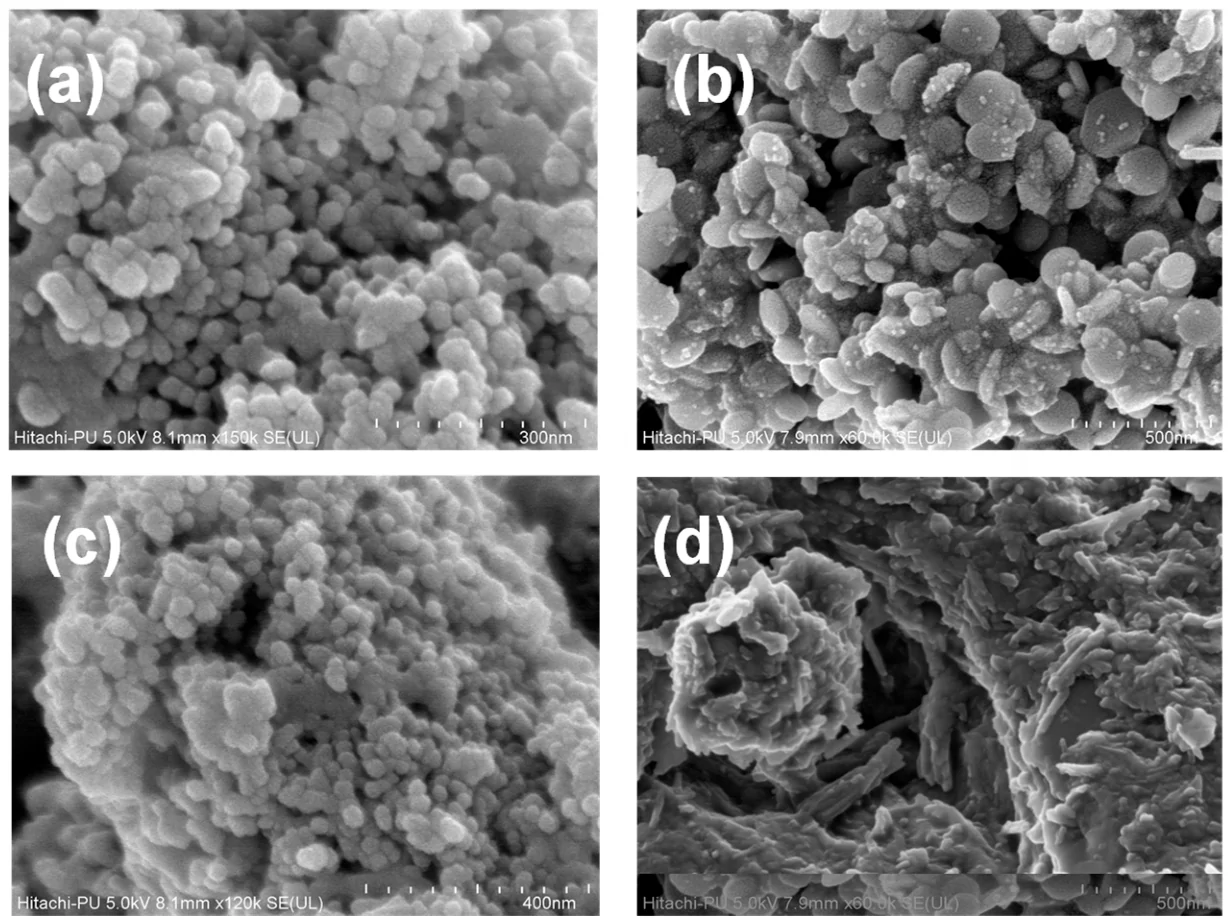
FESEM images of the prepared materials: (**a**) bare Fe_3_O_4_ MNPs, (**b**) CTAB-functionalized MNPs, (**c**) SDS-functionalized MNPs, and (**d**) raw/processed Mill scale, showing the distinct morphology, aggregation behavior, and surface heterogeneity of the materials.

The surface morphology of the prepared materials was investigated by FESEM (Figure 7a–d). The bare Fe_3_O_4_ MNPs (Figure 7a) displayed a predominantly rounded and granular morphology with pronounced agglomeration of the nanoscale particles. Surfactant functionalization resulted in distinct modifications of the surface architecture and aggregation behavior. The CTAB-coated MNPs (Figure 7b) exhibited densely aggregated and interconnected granular domains with a more irregular, corrugated, and heterogeneous surface compared with the bare MNPs, whereas the SDS-coated MNPs (Figure 7c) showed closely packed, rounded nanoparticles forming compact and irregular aggregates with a rough surface texture. These morphological differences suggest that the nature of the surfactant headgroup and its interaction with the Fe_3_O_4_ surface influence nanoparticle organization and interparticle aggregation. The distinct surface architectures generated by CTAB and SDS may consequently modify the interfacial environment, surface charge, and accessibility of surface Fe sites involved in phosphate interaction [31]. The raw/processed Mill scale (Figure 7d) exhibited a highly rugged, coarse, and heterogeneous morphology, comprising large iron-oxide-rich aggregates decorated with irregular flake-, plate-, and elongated rod-like features, which may limit the accessibility of surface-active Fe–OH sites for phosphate [32]. Thus, the FESEM observations demonstrate a clear surfactant-dependent modification of the Fe_3_O_4_ surface morphology, which is consistent with the differences observed in the phosphate- extraction behavior of the functionalized MNPs. The elemental composition of the materials before and after phosphate treatment was further examined by FESEM-EDS (Table 1). The CTAB-MNPs and SDS-MNPs showed no detectable phosphorus before extraction, whereas P appeared after phosphate (Figures S6 and S7) extraction at 0.57 and 0.71 wt. %, respectively. For Mill scale, the P content increased from 0.04 wt.% (due to presence of inherited P_2_O_5_) before treatment to 0.31 wt.% after extraction. The appearance of increase in phosphorus provides complementary evidence for the association of phosphorus-containing species on CTAB-MNPs, SDS-MNPs and Mill scale. Minor changes in the relative Fe and O contents were also observed after treatment, while the characteristic elements associated with the surfactant-functionalized MNPs remained detectable.

### 3.10. XPS Analysis

**Figure 8:**
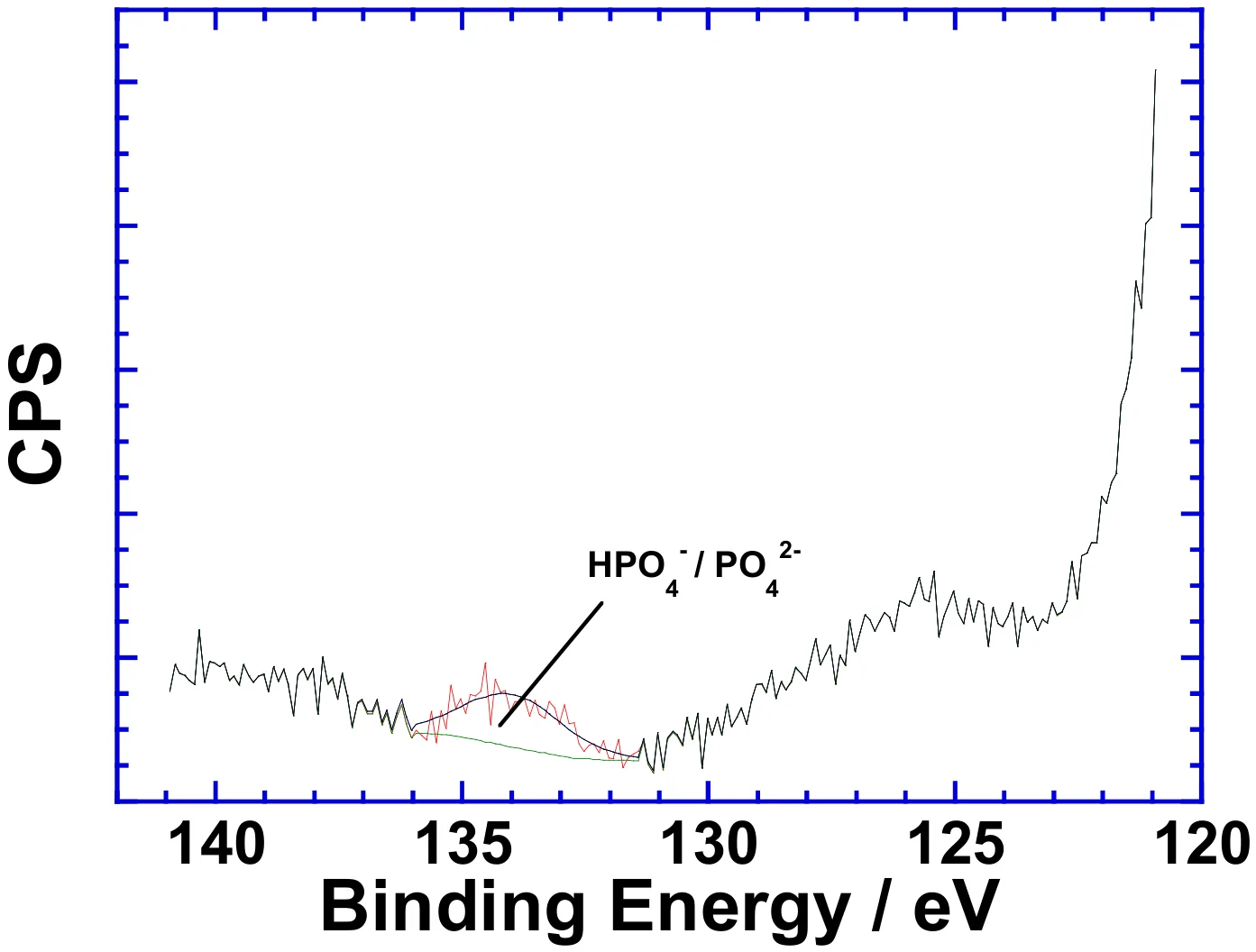
High-resolution XPS spectra of P-2p.

XPS analysis of phosphate-loaded CTAB-MNPs, SDS-MNPs, and Mill scale confirms the surface chemical composition and adsorption mechanism after P extraction (Table S3). The high- resolution Fe 2p spectra consists of Fe^2+^ and Fe^3+^ species corresponding to FeO and Fe_2_O_3_, together with characteristic shake-up satellite peaks, confirming the mixed iron oxide composition of all adsorbents (Figure S8a). CTAB-MNPs and SDS-MNPs also exhibit FeOOH species, indicating the presence of surface hydroxyl groups that provide active Fe–OH sites for P adsorption [33]. The O 1s spectra (Figure S8b) display a major surface component at 531.9–532.2 eV, which is primarily dominated by surface hydroxyls (Fe–OH), phosphate-bound oxygen (P–O), and oxygen-containing surfactant matrices, while the lower binding energy side contains the contributing core lattice oxygen (O^2−^ of iron oxide). The higher binding-energy component at 532.9–534.0 eV corresponds to adsorbed water and organic oxygen species [33]. The C 1s spectra exhibit characteristic components assigned to C–C/C–H, C–O/C–N, C=O, and O–C=O functionalities (Table S3), confirming the presence of surface organic species associated with surfactant modification of in MNPs. A distinct P 2p peak centered at 133.9 eV (Figure 8) confirms successful adsorption of phosphate species on the surface of MNPs. The P atomic % follows the order SDS-MNPs (3 atomic %), Mill scale ≈ CTAB-MNPs (2 atomic %) indicating greater P loading on SDS-MNPs that agrees well with the time- dependent adsorption (Figure 4) and ζ measurements. Thus, XPS clearly supplements P adsorption on the surface of MNPs, which mainly occurs through the formation of Fe–O–P surface complex on the active sites of MNPs.

### 3.11. Mechanism

The extraction of P with CTAB-MNPs, SDS-MNPs, and Mill scale appears to involve interfacial interactions with iron-oxide surface sites together with electrostatic effects, although the relative contribution of these processes differs among the materials. In the model aqueous solution, phosphate species (H_2_PO_4_^−^ and HPO_4_^2−^) can interact with surface hydroxyl groups (Fe–OH) through interfacial ligand-exchange-type interactions, potentially resulting in Fe–O–P surface associations.

Fe–OH + H_2_PO_4_^−^ → Fe–O–PO_3_H + H_2_O

2Fe–OH + HPO_4_^2−^ → (Fe–O)_2_PO_2_H + 2OH^−^

These reactions are presented as possible representations of phosphate interaction with surface Fe–OH groups rather than as definitive evidence of a specific inner-sphere complex. The positively charged CTAB-MNPs (ζ = +19.97 mV) favor electrostatic attraction toward negatively charged phosphate species, which may contribute substantially to P extraction. At the same time, the surfactant layer can influence the accessibility of underlying Fe–OH surface sites and thereby affect the rate of P extraction. In contrast, the negatively charged SDS-MNPs do not favor electrostatic attraction toward negatively charged phosphate species; therefore, their comparatively rapid P extraction suggests that electrostatic attraction is not the sole controlling factor. Instead, accessible iron-oxide surface sites and interfacial diffusion may contribute to the observed P association with SDS-MNPs. Mill scale possesses a heterogeneous, iron-rich oxide surface with exposed surface sites, which may similarly facilitate interaction with phosphate-containing species and account for its observed P extraction behavior. The appearance of phosphorus after treatment in the FESEM-EDS analysis (0.57 wt.% for CTAB-MNPs and 0.71 wt.% for SDS-MNPs), together with the increase from 0.04 to 0.31 wt.% for Mill scale, provides complementary evidence for the association of phosphorus-containing species with the recovered solids. The FTIR and XPS results further support changes in the surface chemical environment following P treatment, while the XRD results indicate that the characteristic crystalline structure of the materials remains essentially unchanged after treatment. Taken together, these observations are consistent with interfacial adsorption involving surface iron-oxide sites and possible Fe–O–P interactions, with electrostatic effects being particularly relevant for the positively charged CTAB-MNPs. However, the present results do not allow inner-sphere Fe–O–P complexation to be established as the sole or predominant mechanism, and contributions from other processes, such as surface precipitation or retention of phosphorus-containing species cannot be completely excluded. Thus, the observed P extraction behavior, SDS-MNPs > Mill scale ≈ CTAB-MNPs, suggests that the accessibility and nature of surface sites, together with interfacial and electrostatic interactions, collectively influence P extraction.

### 3.12. Cost Analysis

A preliminary cost analysis was considered based on the major chemical inputs used for the preparation of the surfactant-functionalized MNPs. The estimated reagent costs were calculated using currently available laboratory-scale market prices for the respective chemicals. Based on the quantities of iron precursors and surfactants used per 20 mL synthesis batch, the estimated direct reagent cost was approximately USD 0.056 per batch for CTAB-MNPs and USD 0.081 per batch for SDS-MNPs, excluding ammonium hydroxide, purification solvents, energy consumption, labor, and equipment-related costs. The current listed prices of the major reagents were used as the basis for this preliminary estimation [34,35]. Although these values represent only the direct reagent cost and should not be considered the final production cost, they provide an initial indication of the material inputs associated with the synthesis. In comparison, Mill scale is an iron-rich industrial residual that can be directly utilized for phosphorus removal without requiring a dedicated nanoparticle synthesis step, making it particularly attractive from a waste valorization and material cost perspective. A comprehensive techno-economic assessment incorporating energy consumption, synthesis yield, purification, solvent recovery, regeneration/reuse, transportation, and scale-up would be required to establish the overall economic feasibility of large-scale application.

## 4. Conclusions

P extraction from agricultural runoff systems was found to be governed by a combination of electrostatic interactions and interfacial surface interactions at the active sites of the iron oxide-based materials. CTAB-MNPs showed a greater contribution from electrostatic attraction between the positively charged surfactant-modified surface and phosphate species, whereas the comparatively rapid P extraction observed with SDS-MNPs and Mill scale indicates a greater contribution from accessible surface-active sites. The FESEM-EDS results further confirmed the appearance of phosphorus on the recovered adsorbents after treatment, while the FTIR, XPS, and XRD results collectively support the association of phosphate-containing species with the iron oxide surfaces without indicating any major alteration in the crystalline structure. The combined results therefore suggest that surface interactions involving Fe–OH sites and the possible formation of Fe–O–P-type surface complexes contribute to P extraction, although other surface-associated processes cannot be completely excluded. Among the investigated materials, SDS-MNPs exhibited the most rapid and efficient P extraction under the applied experimental conditions, followed by Mill scale and CTAB-MNPs. These findings demonstrate the potential of surfactant-functionalized iron oxide nanoparticles and Mill scale as candidate materials for phosphorus extraction from agricultural runoff, while further optimization and independent quantitative validation would be required for practical water-treatment applications.

## Figures and Tables

**Table 1:** Elemental composition (wt.%) of CTAB-functionalized MNPs, SDS-functionalized MNPs, and Mill scale before and after phosphate extraction, as determined by FESEM–EDS analysis.

Element	CTAB (wt.%)		SDS (wt.%)		Mill Scale (wt.%)	
	Before	After	Before	After	Before	After
Fe	42.30	42.09	31.30	32.11	13.15	16.13
O	44.15	41.58	41.20	44.00	37.39	43.87
C	12.20	14.66	26.16	22.02	1.03	0.78
P	0.00	0.57	0.00	0.71	0.04	0.31
N	1.33	1.09	0.12	0.13	2.18	2.66
S	0.02	0.01	1.22	1.03	0.05	0.01
Total	100.00	100.00	100.00	100.00	100.00	100.00

## Data Availability

The data presented in this study are contained within the article and its Supporting Information.

## Supplementary Materials

The following supporting information can be downloaded at: https://www.mdpi.com/article/10.3390/nano16181150/s1, Figure S1: Transmission electron microscopic (TEM) images of CTAB-MNPs (a) and SDS-MNPs (b); Figure S2: UV–Vis titration spectra of a 1 mg P L^−1^; phosphate solution during stepwise addition of SDS-MNPs; Figure S3: Zeta-potential measurements of (a) CTAB-MNPs, (b) SDS-MNPs, and (c) Mill scale; Figure S4: FTIR spectra of pure SDS, KH_2_PO_4_, and P-extracted SDS-MNPs from a real sample of agricultural runoff; Figure S5: FTIR spectra of Mill scale, KH_2_PO_4_, and P-extracted Mill scale from a real sample of agricultural runoff; Figure S6: FESEM-EDS elemental mapping and EDS spectrum of the P-extracted CTAB MNPs; Figure S7: FESEM-EDS elemental mapping and EDS spectrum of the P-extracted Mill scale; Figure S8: XPS high-resolution spectra of Fe 2p (a) and O 1s (b); Table S1: Physicochemical characteristics and reactive phosphorus concentration of the agricultural runoff sample; Table S2a: FTIR peak assignments for CTAB, phosphate species, and P-adsorbed CTAB-MNPs; Table S2b: FTIR peak assignments for pure SDS, KH_2_PO_4_, and P-extracted SDS-MNPs; Table S3: XPS analysis of samples. This information is available free of charge via the internet.
