# Supplementary material for: Functional Magnetic Nanoparticles and Mill Scale for Phosphorus Extraction from Contaminated Water
Source: Nanomaterials (Basel). 2026 Sep 14;16(18):1150. doi: 10.3390/nano16181150 (PMC13610192; doi:10.3390/nano16181150)
Supplement: Supplementary file 1 [file nanomaterials-16-01150-s001.zip › nanomaterials-4485708-supplementary.pdf]

Functional Magnetic Nanoparticles and Mill Scale for Phosphorus Extraction from Contaminated Water

Rajpreet Kaur*, Mandeep Singh Bakshi

Department of Chemistry, Natural and Applied Sciences, University of Wisconsin - Green Bay, 2420 Nicolet Drive, Green Bay, WI 54311-7001, USA.

No. of slides: 14
Figure: 8
Table: 4

Fig S1

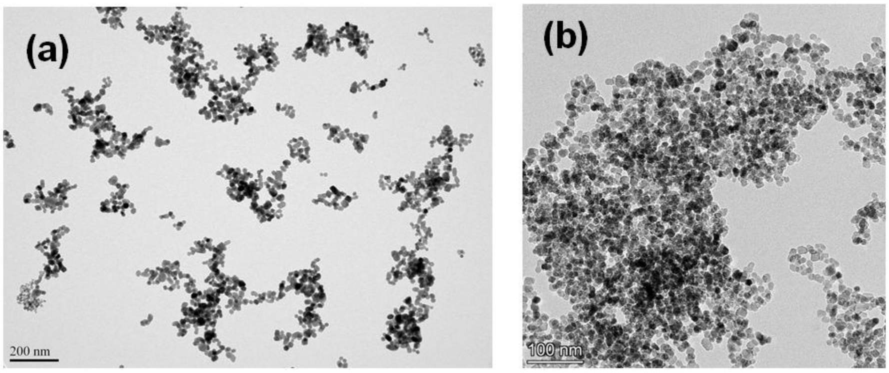

Figure S1. Transmission electron microscopic (TEM) images CTAB-MNPs (a) and SDS-MNPs (b).

Fig S2

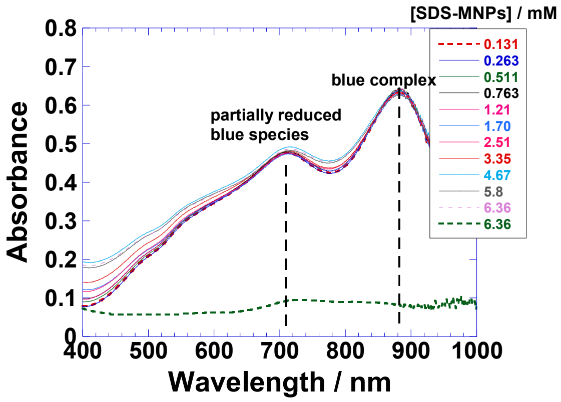

Figure S2. UV–Vis titration spectra of a **1 mg P L^{-1} phosphate solution** during stepwise addition of (SDS-MNPs). Two absorption bands corresponding to molybdenum blue species are observed at ~700 nm and ~880 nm. A little spectral changes during initial titration indicate weak interaction between SDS-MNPs and phosphate ions, while the significant decrease observed after extended interaction (2 days) suggests slow phosphate removal through diffusion-controlled processes (*last green dashed line represents the spectrum taken after 2 days*).

Fig S3

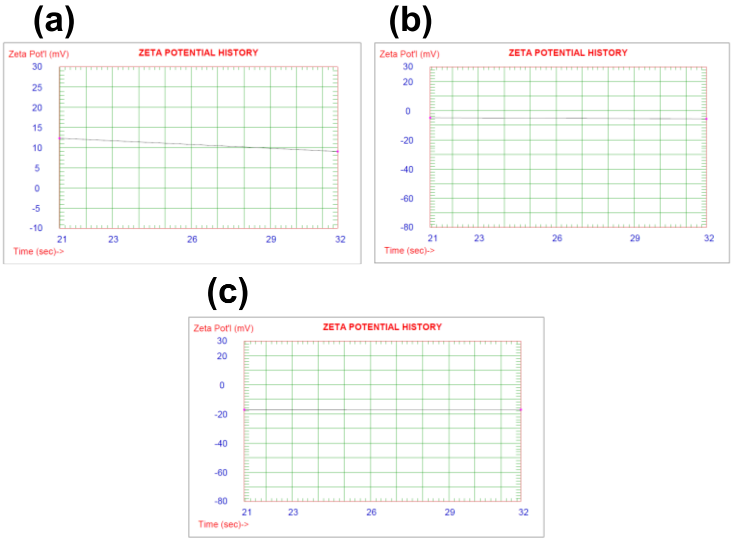

Figure S3. Zeta-potential measurements of (a) CTAB-MNPs, (b) SDS-MNPs, and (c) Mill scale. The measurements demonstrate the distinct surface-charge characteristics of the surfactant-functionalized magnetic nanoparticles and the Mill scale.

Fig S4

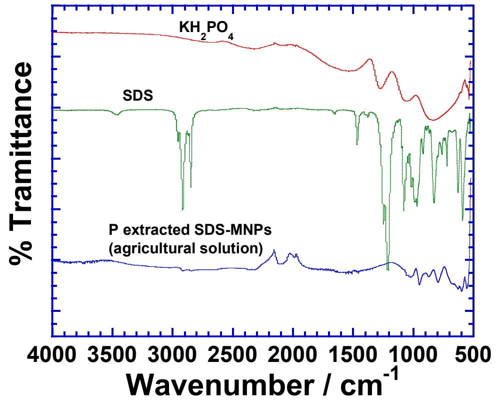

Figure S4. FTIR spectra of pure SDS, KH_2PO_4, P extracted SDS-MNPs from a real sample of agricultural runoff. The appearance of phosphate stretching vibrations in the region of 1000–1100 cm^{-1} together with Fe–O bands near 500–600 cm^{-1} confirms adsorption of P onto SDS-MNPs.

Fig S5

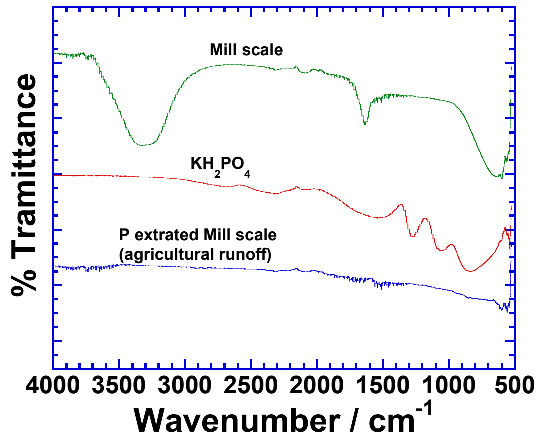

Figure S5. FTIR spectra of Mill scale, KH_2PO_4, P extracted Mill scale from a real sample of agricultural runoff.

Fig S6

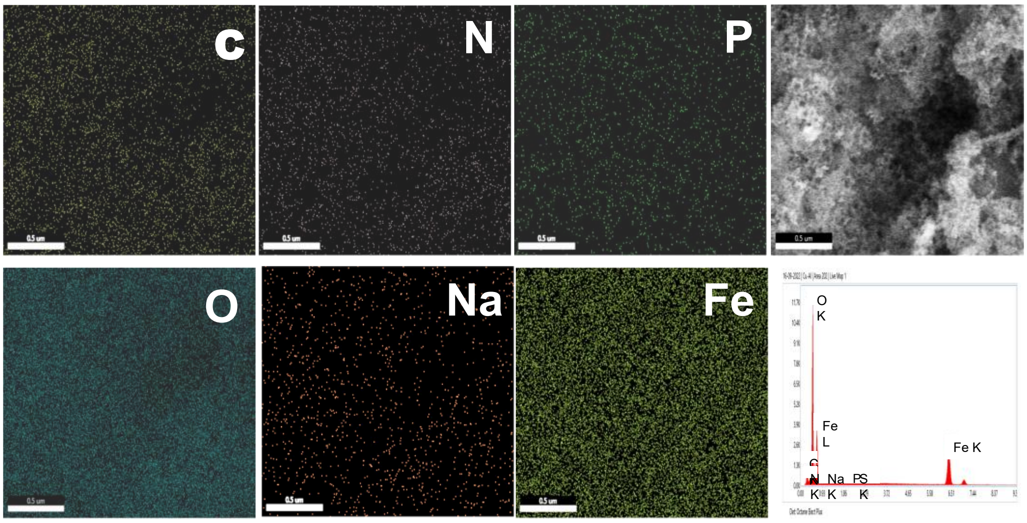

Figure S6. FESEM-EDS elemental mapping and EDS spectrum of the phosphate-treated material, showing the spatial distribution of C, N, P, O, Na, and Fe. The elemental maps demonstrate the presence and distribution of phosphorus on the CTAB MNPs surface after phosphate treatment.

Fig S7

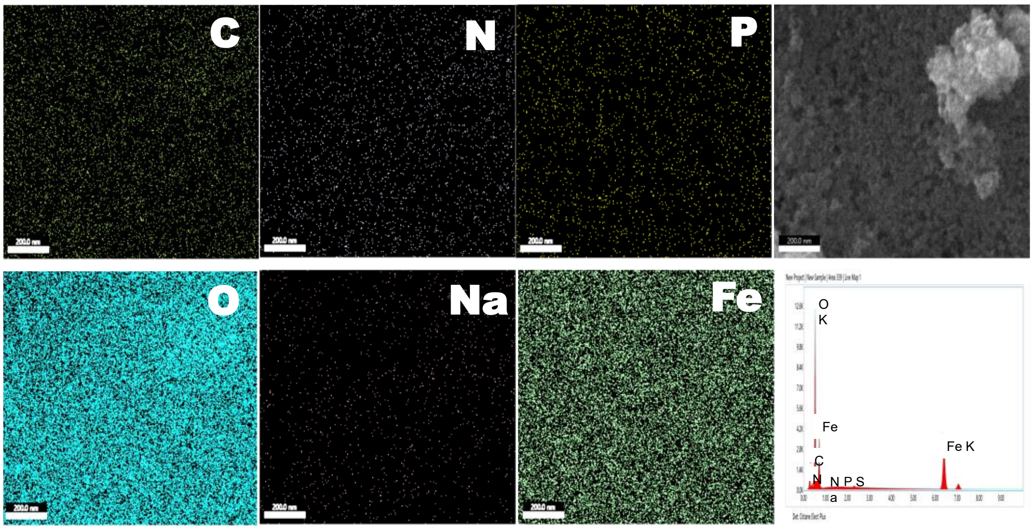

Figure S7. FESEM-EDS elemental mapping and EDS spectrum of the phosphate-treated material, showing the spatial distribution of C, N, P, O, Na, and Fe. The elemental maps demonstrate the presence and distribution of phosphorus on the Mill scale surface after phosphate treatment.

Fig S8

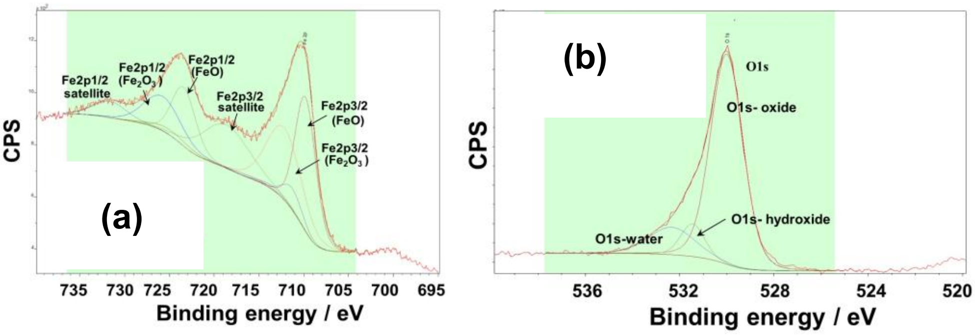

Figure S8. XPS high resolution spectra of Fe 2p (a) and O 1s (b).

Table S1**.** Physicochemical characteristics and reactive phosphorus concentration of the agricultural runoff sample

**Parameter**	**Unit**	**Agricultural runoff**
pH	—	[10.01]
Electrical conductivity	μS cm^{-1}	[204.44]
Untreated reactive phosphorus	mg P L^{-1}	[0.5-0.65]

Table S2a. FTIR peak assignments for CTAB, phosphate species, and P adsorbed CTAB-MNPs.

Pure CTAB (cm^{-1})	After Adsorption (cm^{-1})	Functional Group	Vibrational Mode	Assignment
~3016	~3010	N–CH_3	asymmetric stretching	Quaternary ammonium head group of CTAB
2943	2935–2925	CH_3	asymmetric stretching	Alkyl chain of CTAB
2917	2910–2920	CH_2	asymmetric stretching	Hydrocarbon chain vibration
2870	2860–2855	CH_3	symmetric stretching	Surfactant alkyl chain
2849	2840–2850	CH_2	symmetric stretching	CTAB hydrocarbon chain
1468	1460–1470	CH_2	scissoring	Alkyl chain bending
1394–1360	1370–1385	CH / CH_2	wagging / bending	Surfactant chain deformation
~1080	1040–1050	P–O	stretching	Phosphate tetrahedral vibration
980–900	960–920	P–OH	stretching	Protonated phosphate vibration
~560	~560	Fe–O	stretching	Magnetite nanoparticle core
520–540	520–530	O–P–O	bending	Phosphate bending vibration

Table S2b. FTIR peak assignments for pure SDS, KH_2PO_4 and P extracted SDS-MNPs.

Wavenumber (cm^{-1})	Functional group	Vibrational mode	Assignment
3016	N–CH_3	asymmetric stretching	Quaternary ammonium group of CTAB
2943	CH_3	asymmetric stretching	Alkyl chain vibration
2870	CH_3	symmetric stretching	Surfactant alkyl chain
2917	CH_2	asymmetric stretching	Hydrocarbon chain vibration
2849	CH_2	symmetric stretching	Alkyl chain vibration
1468	CH_2	scissoring	Alkyl chain deformation
1394–1360	CH / CH_2	wagging / bending	Surfactant chain vibration
1000–1100	P–O	stretching	Phosphate tetrahedral vibration
980–900	P–OH	stretching	Protonated phosphate vibration
~560	Fe–O	stretching	Magnetite nanoparticle core

Table S3. XPS analysis of samples. All binding energies/eV are listed in parentheses in the same order in a column below the atomic % and area % entries.

Elements	atomic %	area %	atomic %	area %	atomic %	area %
	P extracted CTAB-MNPs		P extracted SDS-MNPs		P extracted Mill scale	
O1s-1	48.3	67 (531.9)	52	77 (532.1)	68	96 (532.2)
O1s-2	24.4	33 (532.9)	16	23 (533.4)	4	4 (534.0)
C1s (C-C, C-H)	3.4	47 (284.8)	2	31 (284.8)	3	78 (284.8)
C1s (C-OH, C-O-C), (C-N)	2.3	39 (286.3)	1	13 (286.3)	0.4	10 (286.3)
C1s (C=O)	0.4	2 (287.8)	0.1	2 (287.8)	0.6	12 (287.8)
C1s (O-C=O)	1.4	12 (288.8)	2	25 (289.6)		
C1s (CO_3^-)			2	29 (290.0)		
P (PO4-), (HPO4-)	2	100 (133.9)	3	100 (133.9)	2	100 (133.9)
Fe 2p3/2 FeO	16.5	25.1 (709.8)	20.7	24.8 709.9	22	41 709.5
Fe 2p1/2 FeO		12.3 (723.4)		12.2 (723.5)		21 (722.9)
Fe 2p3/2 Fe_2O_3		3.9 (711.3)		4.8 (711.4)		10 (712.6)
Fe 2p1/2 Fe_2O_3		1.9 (724.9)		2.4 (725.0)		5 (726.2)

Table S3: continued

**Fe 2p3/2 FeOOH**		**22.7 (712.2)**		**21.3 (712.3)**		
Fe 2p1/2 FeOOH		11.2 (725.8)		10.5 (725.9)		
Fe 2p3/2 satellite		18.1 (718.7)		19.6 (718.6)		17 (721.8)
Fe 2p1/2 satellite		4.7 (731.7)		4.6 (731.8)		6 734.9
S 2p 3/2			0.5	67 (168.4)		
S2p 1/2			0.4	33 (169.2)		
N 1s	1.3	100 (401.5)				
Na			0.3	100 (1071.8)		
